# Understanding NK cell biology for harnessing NK cell therapies: targeting cancer and beyond

**DOI:** 10.3389/fimmu.2023.1192907

**Published:** 2023-07-18

**Authors:** Eunju Shin, Seong Ho Bak, Taeho Park, Jin Woo Kim, Suk-Ran Yoon, Haiyoung Jung, Ji-Yoon Noh

**Affiliations:** ^1^ Aging Convergence Research Center, Korea Research Institute of Bioscience and Biotechnology, Daejeon, Republic of Korea; ^2^ College of Pharmacy, Chungnam National University, Daejeon, Republic of Korea; ^3^ Department of Functional Genomics, Korea University of Science & Technology (UST), Daejeon, Republic of Korea; ^4^ Immunotherapy Research Center, Korea Research Institute of Bioscience and Biotechnology, Daejeon, Republic of Korea

**Keywords:** natural killer cell, chimeric antigen receptor, immunotherapy, cancer, aging, immune surveillance

## Abstract

Gene-engineered immune cell therapies have partially transformed cancer treatment, as exemplified by the use of chimeric antigen receptor (CAR)-T cells in certain hematologic malignancies. However, there are several limitations that need to be addressed to target more cancer types. Natural killer (NK) cells are a type of innate immune cells that represent a unique biology in cancer immune surveillance. In particular, NK cells obtained from heathy donors can serve as a source for genetically engineered immune cell therapies. Therefore, NK-based therapies, including NK cells, CAR-NK cells, and antibodies that induce antibody-dependent cellular cytotoxicity of NK cells, have emerged. With recent advances in genetic engineering and cell biology techniques, NK cell-based therapies have become promising approaches for a wide range of cancers, viral infections, and senescence. This review provides a brief overview of NK cell characteristics and summarizes diseases that could benefit from NK-based therapies. In addition, we discuss recent preclinical and clinical investigations on the use of adoptive NK cell transfer and agents that can modulate NK cell activity.

## Introduction

Natural killer (NK) cells are innate immune cells that account for 5–10% of peripheral blood (PB) lymphocytes (1). NK cells are classified as cytotoxic lymphocytes that play a crucial role in the recognition and elimination of malignant or infected cells. The expression of major histocompatibility complex class I (MHC-I) molecules in cells is often suppressed by neoplastic transformation and viral infection, making these cells “missing self” targets for NK cells. This mechanism allows NK cells to overcome suppressed immunosurveillance by CD8^+^ T cells, which require antigen presentation on MHC-I molecules (2). Unlike T cells, NK cells can be primed with interleukin-2 (IL-2) or IL-15 without licensing. Mature NK cells express various receptors that modulate their activity against target cells. These unique characteristics make NK cells an attractive option for adoptive immune cell therapy. Allogeneic transfer of NK cells derived from healthy donors or the use of chimeric antigen receptor (CAR)-NK cells has shown promising results for the treatment of various types of cancers. In addition, there is growing interest in exploring the use of NK cell therapies for other indications, such as viral infections or aging. Several therapeutic strategies have been developed to enhance NK cell function. This review provides an overview of the NK cell mechanisms and recent advancements in NK cell therapy. The first part describes these pathways during the developmental process and the intracellular signaling pathways. The second section discusses the application of NK cell therapies to various diseases, followed by an introduction to the genetic engineering of NK cells in the third section. Finally, the fourth section covers approaches that can stimulate NK cells, which may be combined with genetic engineering for NK cell therapies in the future.

## NK cell biology

### NK cell development

NK cells originate from common lymphoid progenitors (CLPs) that are derived from hematopoietic stem cells (HSCs) in the bone marrow (BM) (3). Unlike T and B cells, which generate antigen receptors via gene rearrangements, NK cells express a broad range of germline-encoding receptors (4). The development of NK cells is facilitated by cytokine IL-15, which binds to the IL-15 receptor subunit alpha, CD122 (IL-2/IL-15 receptor subunit beta), and the common gamma chain on NK progenitors (NKPs), resulting in the commitment of these cells to the NK cell lineage (5). Although IL-2 promotes NK cell proliferation during *ex vivo* culture, it is not essential for the development of NK cells in mice (6). NKPs differentiate into immature NK cells (iNKs), which are identified by the expression of NK1.1 marker in mice and the NKp46 activation receptor in both mice and humans (7). In humans, CD56^bright^ NK cells are abundant in secondary lymphoid tissues and mature into CD56^dim^ NK cells in PB (8). Usually, CD56^dim^ NK cells are more cytotoxic and express the CD16 (Fc gamma RIIIA) receptor, while CD56^bright^ NK cells produce more cytokines and have lower levels of CD16 expression (9). CD56^dim^ NK cells are generally considered as a more mature form of NK cells (mNK); however, the precise mechanism governing the differentiation of these two subsets is not yet well understood. NK cells can be classified into group 1 innate lymphoid cells (ILCs), as they produce type 1 cytokines, interferon gamma (IFN-γ) and tumor necrosis factor (TNF) (10–13). NK cells also have tissue-specific subsets with distinct functions that potentially modulate immune responses in different contexts (14–16).

**Figure 1:**
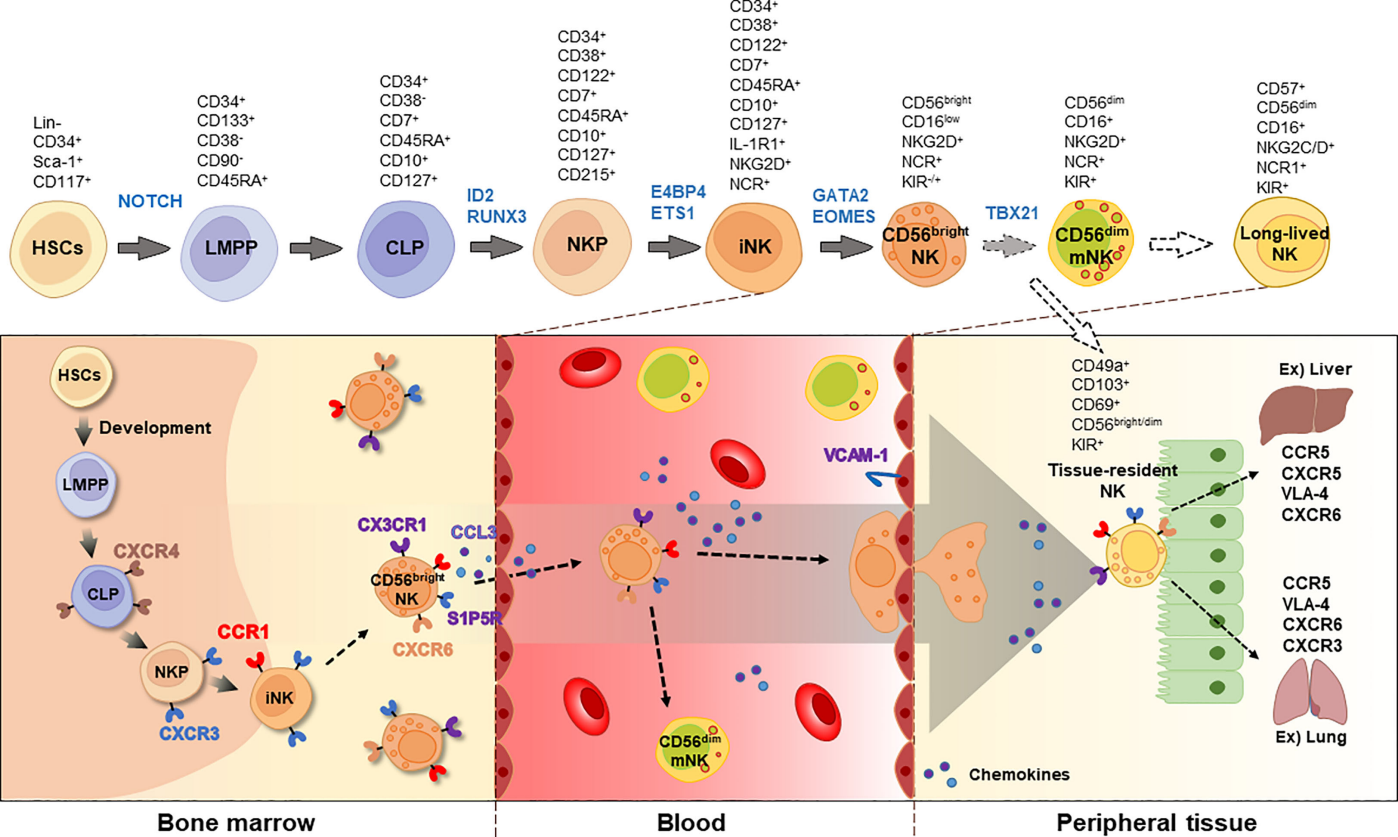
Developmental process of NK cells. NK cells originates from HSCs and CLPs in the bone marrow. The immature NK cells express CD122 and NCRs, such as NKp46, NKp30, and NKp44. Chemokine receptors, including CXCR3, CX3CR1, and S1P5R, are involved in the egression of NK cells. In the blood, two types of NK cells are majorly found, CD56^bright^ and CD56^dim^, with CD56^dim^ NK cells expressing CD16. Long-lived NK cells can be distinguished by increased expression of CD57. Tissue-resident NK cells express CD49a, CD103, CD69, and CD56. The blocked arrows with dotted lines suggest that further research is required to fully understand these processes. *HSC*, hematopoietic stem cell; *LMPP*, lympho-myeloid primed progenitor; *CLP*, common lymphoid progenitor; *NKP*, NK progenitor; *iNK*, immature NK; *mNK*, mature NK; *NCR*, NK cell receptor; *KIR*, killer cell immunoglobulin-like receptor; *VLA-4*, very late antigen-4.

NK cell development is regulated by several transcription factors including *E4BP4*, *TBX21*, *EOMES*, *GATA3*, and *ID2* (17). For instance, mice lacking *E4bp4* exhibit decreased levels of NK1.1^+^ NKPs and impaired NK cell-mediated cytotoxicity (18). In mice, CD27^+^ NK cells mature into CD27^-^CD11b^high^ NK cells. A deficiency of *Aiolos* (*Ikzf3*), a member of the Ikaros family of zinc-finger transcription factors, was found to prevent the maturation of CD27^+^ to CD27^-^ NK cells, while proliferation of NK1.1^+^CD122^+^ iNKs was enhanced in *Aiolos*-deficient cells following IL-15 stimulation (19). Likewise, researchers have investigated the gene network involved in NK cell development and the regulation of NK cell function. Detailed markers and the distribution of NK cells during development are shown in **Figure 1**.

### NK cell receptors and functions

NK cells eliminate target cells that appear to be missing self or stressed, such as those infected with viruses, cancer, or those undergoing cellular senescence. NK cell killing can be achieved through the activation of receptors such as NKG2D and natural cytotoxic receptors (NCRs) NKp30, NKp44, and NKp46. Ligands for these receptors include major histocompatibility complex class I (MHC-I) chain-related polypeptide A (MICA) and MICB proteins, a family of six cytomegaloviral unique long 16 (UL16)-binding proteins (ULBP 1-6) (20–22), or murine retinoic acid early transcript 1 (RAET1), histocompatibility H60 (H60), and murine UL16-binding protein-like transcript 1 (MULT1), and antigens from pathogens (23–25). DNAX accessary molecule 1 (DNAM1) and signaling lymphocytic activation molecule (SLAM) family receptors, including 2B4, SLAMF7, and NKp80, act as co-receptors to enhance NK cell activity (26). Upon activation, NK cells use an immunoreceptor tyrosine-based activation motif (ITAM) or tyrosine-based signaling motif (YINM) to activate receptors to stimulate protein kinases such as SYK and ZAP70, resulting in the secretion of perforins and granzymes (27). This process leads to apoptosis of the target cells. Furthermore, CD56^dim^ NK cells in PB can mediate antibody-dependent cellular cytotoxicity (ADCC) via the Fc gamma receptor CD16.

The killer cell immunoglobulin-like receptor (KIR) family and CD94/NKG2A heterodimer are inhibitory receptors on NK cells that recognize MHC Class I molecules (28). Humans have 14 KIR genes specific to each individual (29). KIRs are composed of two or three immunoglobulin-like (Ig-like) domains: KIR2D and KIR3D. KIRs have either activating or inhibitory functions. Generally, KIRs with a short cytoplasmic tail are activated, while those with long cytoplasmic tails are inhibitory. Activating KIRs, such as KIR2DS1, KIR2DS2, KIR2DS3, KIR2DS4, KIR2DS5, and KIR3DS1 associate with the transmembrane adaptor DAP12 to transduce activation signals in NK cells. Inhibitory KIRs, such as KIR2DL1, KIR2DL2, KIR2DL3, KIR2DL5, KIR3DL1, KIR3DL2, and KIR3DL3 have an immunoreceptor tyrosine-based inhibitory motif (ITIM) (30). PB NK cells have a diverse repertoire owing to the random expression of KIR genes in each NK cell, resulting in various NK cell clones with distinct receptor expression patterns. Consequently, a subset of NK cell clones may recognize a unique ligand expressed by a particular tumor cell (31, 32). Inhibitory receptors on NK cells include KLRG1, SIGLEC7, SIGLEC9, leukocyte immunoglobulin-like receptor subfamily B member 1 (LILRB1), CD161, TIGIT, LAG3, TIM3, and PD-1, which recruit protein tyrosine phosphatases, such as SHIP1 (also known as PTPN6) and SHP2 (also known as PTPN11), to eliminate tyrosine phosphorylation (27).

NK cells produce chemokines and express various receptors. Resting CD56^bright^ NK cells preferentially express chemokine receptors related to BM or lymph node residency, such as CCR2, CCR7, CXCR3, and CXCR4. In contrast, CD56^dim^ NK cells primarily express CX3CR1, ChemR23 or CXCR1 (33, 34). CXCR1 is a cognate receptor of CXCL8, a senescence-associated secretory phenotype (SASP) factor that can also be released from NK cells (35, 36).

### Signal transduction pathways in NK cell activation

Upon recognition of NK cell activation receptors, the phosphorylation of the adaptor proteins DAP10 and DAP12 initiates downstream signal transduction (37, 38). The activation of phosphoinositide 3-kinase (PI3K) plays a crucial role in NK cells by recruiting AKT (also known as protein kinase B), which promotes cell survival and metabolism (39, 40). The MAPK pathway is also activated, leading to sequential phosphorylation events involving extracellular signal-regulated kinase (ERK), c-Jun N-terminal kinase (JNK), and p38, ultimately resulting in gene transcription and cytokine secretion (41–43). Phospholipase Cγ2 (PLCγ2) generates inositol 1,4,5-triphosphate (IP3) and diacylglycerol (DAG), which trigger calcium ion release from the endoplasmic reticulum and activate protein kinase C (PKC). Increased intracellular calcium levels stimulate the Ca^2+^/calmodulin (CaM)-dependent phosphatase calcineurin, which dephosphorylates nuclear factor of activated T cells (NFAT). Dephosphorylated NFAT translocates into the nucleus, promoting the transcription of genes involved in NK cell cytotoxicity and cytokine production, as well as inducing NK cell development by inducing *EOMES* and *TBX21* upregulation (44).

**Figure 2:**
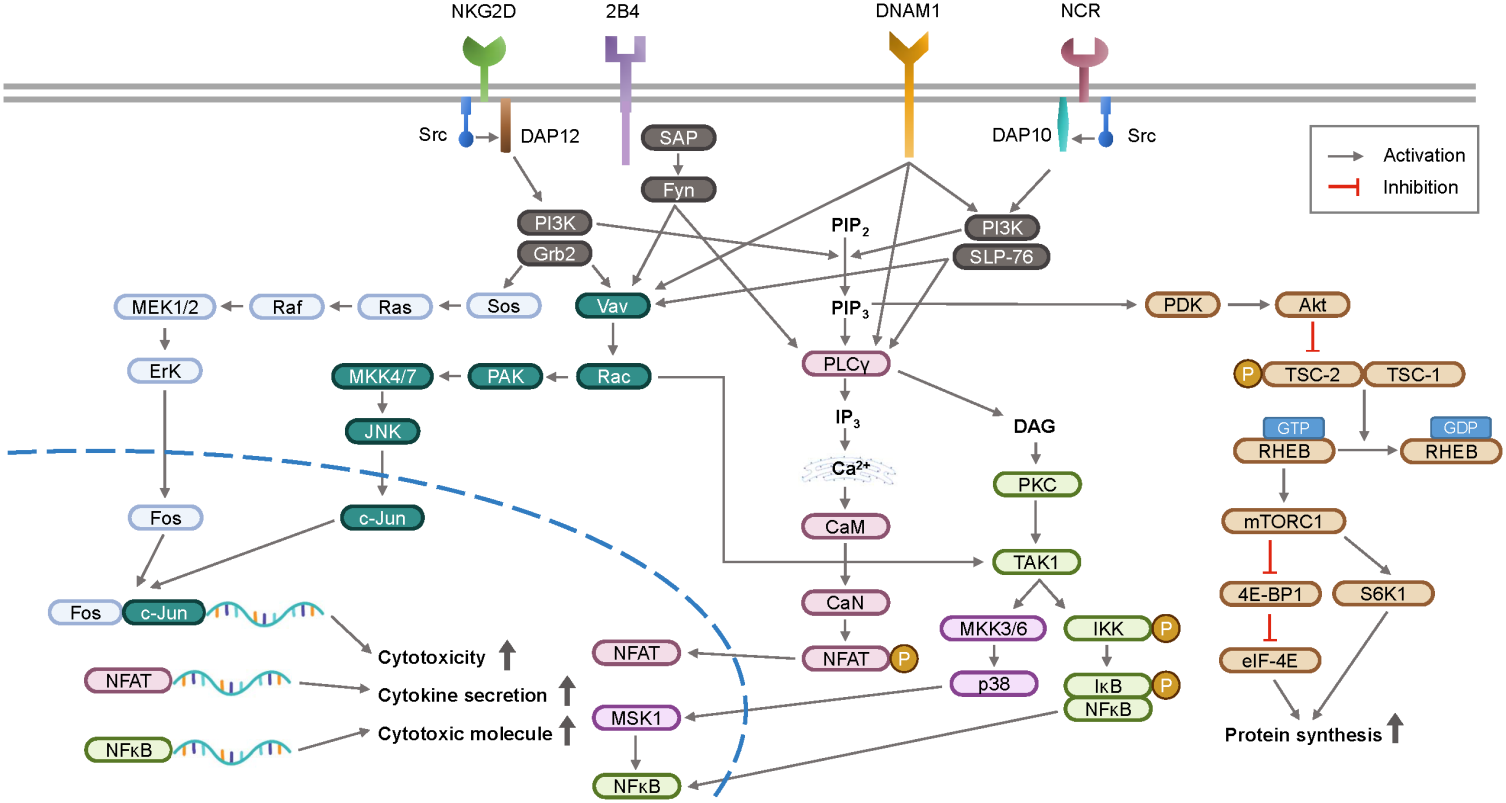
A schematic overview of signal transduction pathway for NK cell activation. Upon recognition of ligands, activation receptors on NK cell surface initiate intracellular signaling via adaptor proteins DAP10 and DAP12. These signaling pathways stimulate the transcription of genes involved in cytokines and cytotoxicity, which are key functions of NK cell surveillance. *DAP12*, DNAX-activating protein of 12 kDa; *DAP10*, DNAX-activating protein 10; *SAP*, slam-associated protein; *PI3K*, phosphoinositide 3-kinase; *Grb2*, growth factor receptor-bound protein 2; *PAK*, p21-activated kinase; *JNK*, c-Jun N-terminal kinase; *SLP-76*, Src homology 2 domain-containing leukocyte protein of 76 kDa; *PIP_2_*, phosphatidylinositol 4,5-bisphosphate; *PIP_3_*, phosphatidylinositol 3,4,5-triphosphate; *PLCγ*, phospholipase C γ; *IP_3_*, inositol 1,4,5-triphosphate; *CaM*, calmodulin; *CaN*, calcineurin; *NFAT*, nuclear factor of activated T-cells; *DAG*, diacylglycerol; *PKC*, protein kinase C; *MSK1*, mitogen and stress activated protein kinase-1; *IKK*, IκB kinase; *IκB*, inhibitor of NFκB; *NFκB*, nuclear factor kappa B; PDK, phosphoinositide dependent kinase; Akt, protein kinase B; TSC, tuberous sclerosis complex; *RHEB*, ras homologue enriched in brain; *mTORC1*, mammalian target of rapamycin complex 1; *4E-BP1*, eIF4E-binding protein 1; *eIF-4E*, eukaryotic initiation factor 4E; *S6K1*, ribosomal protein S6 kinase beta-1.

The mechanistic/mammalian target of rapamycin (mTOR) signaling pathway is upregulated by cytokines or growth factors, and inhibition of mTOR suppresses NK cell activity (45, 46). In the immune suppressive tumor microenvironment, TGF-β1 has been found to inhibit IL-15-promoted NK cell activation via the mTOR pathway (47). Upon activation, NK cells release cytotoxic granules containing perforin and granzymes. Granzymes induce target cell apoptosis and perforin forms pores in the target cell membrane. Downstream signaling pathways of NK cell receptors activate transcription factors, such as nuclear factor-kappa B (NF-κB) (48) and STAT4, promoting IFN-γ gene transcription. IFN-γ is secreted by activated NK cells in response to viral infections or cell transformation. The signaling pathways involved in NK cell activation are summarized in **Figure 2**.

### NK cell characteristics in the elderly

Immunosenescence, which typically involves a decrease in the frequency of naïve T and B cells, and an increase in the number of effector/terminally differentiated memory cells, has been examined and discussed in many studies. Although the number of NK cells tends to remain stable or increase with age, their function seems to be impaired (49). Although IL-2 stimulates NK cell responsiveness, cells from older donors are less activated than those from younger donors (50). Notably, NK cell dysfunction has been linked to the development of diseases, particularly an elevated risk of cancer with age (51–53).

The age-related decline in NK cell activity can be attributed to several intrinsic and extrinsic factors. Although changes in perforin levels and KIR diversity have been investigated, other mechanisms may be involved (54, 55). Human NK cells exhibit telomere shortening with age in both the CD56^bright^ and CD56^dim^ populations (56). Extrinsically, research has focused on changes in endocrine factors with age, as some appear to be associated with NK cell function (57–59). For example, glucocorticoids, which inhibit NK cell function by reducing perforin and granzyme B levels (60, 61), are upregulated in humans (62). Additionally, the plasma level of TGF-β1 increases as senescent fibroblasts secrete more of it (63).

Recently, the coronavirus disease 2019 (COVID-19) pandemic has highlighted advanced age as a major risk factor for mortality, with 95% of deaths occurring individuals over 50 years of age (64). This suggests that immunosenescence in the elderly population may partially play a role in determining the severity of viral infections (65, 66). Several studies have used clinical samples to investigate these mechanisms. For instance, it has been shown that type I IFN signaling is increased in mild cases of COVID-19, while impaired in severe cases (67). Single-cell RNA sequencing (scRNA seq) revealed a decrease in the expression of the human leukocyte antigen-DR isotype (HLA-DR) on monocytes in patients with moderate or mild COVID-19 compared to healthy controls (68). Notably, reduced numbers and cytolytic activity of NK cells have been associated with severe COVID-19 (69). In a study comparing severely young and old immune cells, it was found that the functional exhaustion of NK cells was more pronounced in older individuals (70).

The level of CD57 on NK cells is a marker commonly used to measure the number of long-lived NK cells in the blood, with higher levels typically observed in older individuals (**Figure 1**). These populations were also considered less cytotoxic. However, CD57 expression levels can vary depending on infection, chronic diseases, and age. Furthermore, NKG2C^+^CD57^+^ NK cells have been suggested to have strong and rapid antiviral and antitumor effects, indicating an adaptive immune response (71–73). Killer cell lectin-like receptor subfamily G member 1 (KLRG1) is an inhibitory receptor found in terminally differentiated effector lymphocytes, including memory CD8^+^ T and NK cells. It has been shown that an increase of KLRG1^+^CD57^+^ NK cells was induced by COVID-19 infection, particularly in older individuals, indicating NK cell exhaustion (70). Therefore, the significance of CD57^+^ NK cells should be interpreted in the context of disease and their function as long-lived and/or memory NK cell populations.

## Targetable diseases by NK cell therapies

Researchers have found that HLA haplotype-mismatched grafts with KIR ligand incompatibility can greatly reduce relapse in post-transplant acute myeloid leukemia (AML) (74). Moreover, T cell-depleted allogeneic hematopoietic stem cell transplantation (alloHSCT) is a beneficial therapeutic approach for cancer (75). Adoptive transfer of NK cells is a safer option than CAR-T cell therapy for cytokine release syndrome (CRS) or neurotoxicity (76, 77). This can also reduce costs. This section focuses on diseases that have been targeted by adoptive NK cell therapies, including CAR-NK cells, and potential target diseases. The list of CAR-NK cells that are under development for the treatment of diseases, such as solid tumors and hematologic malignancies, is displayed in **Table 1**.

**Table 1:** A list of current strategies of CAR-NK cell therapy, in combination with other treatments.

Genetic Target	Cell type	Combination (Strategy)	Target Disease	Clinical Trial Phase	Trial Status	Clinical Trial register	Ref
PSMA	NK	Chemotherapy	Prostate cancer	Phase 1	Recruiting	NCT03692663	
NKG2D-ACE	NK	N/A	COVID-19	Phase 1,2	Unknown	NCT04324996	
ROBO1	NK	N/A	Malignant tumor	Phase 1,2	Unknown	NCT03931720	
ROBO1	NK-92	N/A	Solid tumor	Phase 1,2	Unknown	NCT03940820	
ROBO1	NK-92	N/A	Pancreatic cancer	Phase 1,2	Unknown	NCT03941457	(78)
HER2	NK-92	N/A	Glioblastoma	Phase 2	Recruiting	NCT03383978	(79)
MUC1	NK-92	N/A	Lung cancer Pancreatic cancer Gastric cancer Breast Cancer Colorectal cancer Glioma	Phase 1,2	Unknown	NCT02839954	
CCCR (PD-1, NKG2D, 41BB)	NK-92	N/A	Lung cancer	Phase 1	Enrolling by invitation	NCT03656705	(80)
PD-L1	haNK	N/A	Advanced Solid tumor Metastatic tumor	Phase 1	Active, Not recruiting	NCT04050709	
PD-L1	haNK	Chemotherapy	Lung cancer Colorectal cancer Gastric cancer Cervical cancer Carcinoma model Melanoma	Phase 2	Active, Not recruiting	NCT03228667	
PD-L1	haNK	Chemotherapy	Gastroesophageal junction cancer Advanced head and neck squamous cell carcinoma	Phase 2	Recruiting	NCT04847466	
PD-L1	haNK	Chemotherapy Radiation therapy	Pancreatic cancer	Phase 2	Recruiting	NCT04390399	
Mesothelin	PB-NK	N/A	Ovarian cancer	Phase 1	Unknown	NCT03692637	
NKG2D	PB-NK	N/A	Adult solid tumor	Phase 1	Unknown	NCT03415100	(81)
N/A	iPSC-NK (FT500)	Chemotherapy	Solid tumor Lymphoma	Phase 1	Completed	NCT03841110	
hnCD16	iPSC-NK (FT516)	Chemotherapy	Adult solid tumor	Phase 1	Completed	NCT04551885	
N/A	INTK	N/A	B-cell leukemia B-cell lymphoma	Phase 1,2	Unknown	NCT04747093	
CD19	NK	N/A	B-cell lymphoma	Phase 1	Unknown	NCT02944162	
CD19	NK	N/A	Non-Hodgkin lymphoma	Phase 1	Active, Not yet recruiting	NCT04639739	
CD22	NK	N/A	B-cell lymphoma	Phase 1	Unknown	NCT03692767	
CD19/CD22	NK	N/A	B-cell lymphoma	Phase 1	Unknown	NCT03824964	
CD33/CLL1	NK	N/A	Acute myeloid leukemia	Phase 1	Recruiting	NCT05215015	
CD33	NK	Chemotherapy	Acute myeloid leukemia	Phase 1	Recruiting	NCT05008575	
CD19	NK	N/A	Lymphocytic leukemia Non-Hodgkin lymphoma	Phase 1	Recruiting	NCT05410041	
CD7	NK-92	N/A	Leukemia Lymphoma	Phase 1,2	Unknown	NCT02742727	
CD19	NK-92	N/A	Leukemia Lymphoma	Phase 1,2	Unknown	NCT02892695	
CD33	NK-92	N/A	Acute myeloid leukemia	Phase 1,2	Unknown	NCT02944162	(82)
BCMA	NK-92	N/A	Multiple myeloma	Phase 1,2	Unknown	NCT03940833	(83)
CD19	PB-NK	N/A	Non-Hodgkin lymphoma	Phase 1	Recruiting	NCT04887012	
NKG2D	PB-NK (Haploidentical donor)	N/A	Acute myeloid leukemia Myelodysplastic syndrome	Phase 1	Unkonwn	NCT04623944	
CD19	PB-NK	N/A	Acute lymphoblastic leukemia	Phase 1	Completed	NCT00995137	
CD19	PB-NK	N/A	Lymphoma Lymphocytic leukemia Lymphoblastic leukemia Macroglobulinemia	Phase 1	Recruiting	NCT05020678	
CD19	PB-NK (Allogenic donor)	Chemotherapy	B-cell lymphoma B cell acute lymphoblastic leukemia	Phase 1	Recruiting	NCT05379647	
CD19/iCas9/IL-15	CB-NK	Chemotherapy	B-cell lymphoid malignancy Lymphocytic leukemia Non-Hodgkin lymphoma	Phase 1,2	Complete	NCT03056339	
CD5	CB-NK	Chemotherapy	Hematological malignancy	Phase 1	Active, not yet recruiting	NCT04796675	
CD19	CB-NK	Chemotherapy	Lymphocytic leukemia Non-Hodgkin lymphoma	Phase 1	Recruiting	NCT04796675	
BCMA	CB-NK	Chemotherapy	Refractory multiple myeloma	Phase 1	Recruiting	NCT05008536	
CD19/IL-15	CB-NK	Chemotherapy	B-cell non-Hodgkin lymphoma	Phase 2	Recruiting	NCT05020015	
CD70/IL-15	CB-NK	Chemotherapy	B-cell lymphoma B-cell malignancy Non-Hodgkin lymphoma	Phase 1,2	Recruiting	NCT05092451	
BCMA	iPSC-NK	Chemotherapy	Multiple myeloma Myeloma	Phase 1	Recruiting	NCT05182073	
CD19/EGFR/IL-15	iPSC-NK	N/A	B-cell malignancy Non-Hodgkin lymphoma	Phase 1	Recruiting	NCT05336409	
hnCD16	iPSC-NK (FT516)	Chemotherapy	Acute myelogenous leukemia B-cell lymphoma	Phase 1	Recruiting	NCT04023071	
hnCD16/IL-15/CD38 KO	iPSC-NK (FT538)	Chemotherapy	Acute myeloid leukemia Multiple myeloma Myeloma	Phase 1	Recruiting	NCT04614636	(84)
hnCD16/IL-15/CD38 KO	iPSC-NK (FT538)	Chemotherapy	Acute myeloid leukemia Multiple myeloma Monocytic leukemia	Phase 1	Recruiting	NCT04714372	
CD19/hnCD16/IL15R	iPSC-NK (FT596)	Chemotherapy	Acute myeloid leukemia Monocytic leukemia	Phase 1	Recruiting	NCT04714372	(85)
CD19/hnCD16/IL15R	iPSC-NK (FT596)	Chemotherapy	Acute myeloid leukemia Multiple myeloma	Phase 1	Recruiting	NCT04614636	

N/A, Not applicable; PSMA, prostate Specific Membrane Antigen; NKG2D, NKG2-D Type II Integral Membrane Protein; ACE, angiotensin-Converting Enzyme; ROBO1, Roundabout, Axon Guidance Receptor, Homolog 1; HER2, human epidermal growth factor receptor 2; MUC1, mucin-1; CCCR, chimeric costimulatory converting receptor; PD1, programmed cell death protein 1; PD-L1, programmed cell death ligand 1; hnCD16, high-affinity noncleavable variant of CD16a; CLL1, C-Type lectin-like molecule-1, BCMA, B-cell maturation antigen; iCAS9, inducible caspase 9; EGFR, epidermal growth factor receptor; IL-15(R), Interleukin 15(receptor); KO, knockout; haNK, high affinity NK cell, NK-92 cells engineered to express the high affinity CD16; PB-NK, peripheral blood derived NK cell; CB-NK, cord blood derived-NK cell; iPSC-NK, inducible pluripotent stem cell derived-NK cell; ITNK, induced-T-to-natural killer cell.

### Hematologic malignancies

Hematologic malignancies, including AML, chronic lymphocytic leukemia (CLL), lymphoma, and multiple myeloma (MM), have become targetable diseases by adoptive immune cell therapies (86, 87). These therapies are effective because the administered immune cells can efficiently travel to tumor site in hematopoietic organs (88). In addition, hematopoietic tumor cells often express relatively homogeneous antigens such as CD19 in diffuse large B-cell lymphoma (DLBCL) and B-cell maturation antigen (BCMA) in MM, making it easier to apply CAR or monoclonal antibody (mAb) therapies. Since its approval by the US Food and Drug Administration (FDA) in 2017, six CAR-T cell therapies have been developed. Four of these target CD19, including the first CAR-T cell therapy and the remaining target, BCMA (89, 90). They are used to treat refractory or relapsed B cell ALL, B cell non-Hodgkin’s lymphoma, follicular lymphoma, mantle cell lymphoma, and MM (91). Notably, a phase I/II clinical trial of anti-CD19 CAR-transduced cord blood-derived NK cells showed promising results, with a 73% response rate and long-term survival of infused CAR-NK cells without CRS toxicity (92). CAR-NK cells have the potential to benefit from the intrinsic characteristics of NK cell-activating receptors, which may maintain therapeutic efficacy even after the CAR is lost (93). As a result, CAR-NK cells are being developed for hematological malignancies such as T-cell ALL, and preclinical studies have shown the effectiveness of CAR-NK cells specific for antigens such as CD5 and CD7 (94, 95).

### Solid tumors

#### Primary solid tumors

Various studies have shown that a higher number of NK cells in the PB is associated with better outcomes in solid tumors such as non-small cell lung cancer (NSCLC) (96), melanoma (97), and colorectal cancers (98). Similarly, increased tumor-infiltrating NK cells are related to better prognosis in solid tumors, such as hepatocellular carcinoma (HCC) (99), prostate cancer (100), and renal cell carcinoma (101). However, the function of NK cells is often impaired by the tumor microenvironment, which leads to a significant decrease in both the frequency and function of NK cells in solid tumors (102–105). It was demonstrated that NK cells exhibit lower levels of cytokines like IFN-γ and TNFα in gastric cancer (106). Studies have also found that NK cells had higher expression of inhibitory receptors while showing decreased levels of activating receptors in breast cancer or pancreatic cancer samples (107). Furthermore, the presence of the soluble form of B7-H6, a ligand of NKp30, has been associated with reduced NKp30 expression in NK cells, which may contribute to their suppression (108, 109). Markers of exhaustion such as PD-1 (110, 111), TIM3 (112), and TIGIT (113), which have been studied in T cells, are also expressed in NK cells from cancer patients, indicating an exhaustive phenotype in NK cells similar to that in T cells (113).

Among peripheral tissues, the lungs are rich in NK cells, comprising 10–20% of lung lymphocytes (114, 115). Lung cancer is a significant form of cancer, as it is one of the most frequently diagnosed cancers and remains a leading cause of cancer-related deaths (116, 117). Therefore, the anti-tumor effects of NK cells against lung cancer have been suggested (118, 119). As a therapeutic approach for treating NSCLC, allogeneic NK cells obtained from healthy donors were combined with pembrolizumab, a PD-1 inhibitor. The results of this study suggest that the addition of allogeneic NK cells enhances the anti-tumor immune function of pembrolizumab. The study showed a reduction in PD-1 levels in PB NK cells, an increase in IFN-γ, an increased proportion of NK cells, and a significant decrease in circulating tumor cells (CTCs) (120).

The phenotype of NK cells in the lungs is mostly CD56^dim^CD16^+^, which is similar to that in PB but distinct from those in other organs, such as the liver and secondary lymphoid organs (121). Although PB-derived CD56^dim^CD16^+^ NK cells demonstrate greater differentiation and target cell-killing effects, lung NK cells exhibit weaker responses to target cells (122). In contrast, CD56^bright^ NK cells in the lung co-express CD49a, CD103, and CD69, indicating they are tissue-resident NK cells (123, 124). Furthermore, NKG2C^+^ adaptive-like NK cells have been detected in both the blood and lungs, with a subset of CD49a^+^KIR^+^NKG2C^+^CD56^bright^CD16^-^ lung NK cells demonstrating heightened responsiveness to target cells (125). Therefore, these NK cell subsets may be potential candidates for novel anti-tumor NK cell therapies.

The liver receives a substantial amount of blood flow and encounters numerous foreign antigens. It harbors a diverse range of immune cells, with NK cells comprising approximately half of all hepatic lymphocytes (126). HCC is a highly prevalent malignancy worldwide and the second leading cause of cancer-related mortality (127). Studies have indicated a notable correlation between a decrease in the proportion of NK cells producing IFN-γ and the severity of HCC, as well as an increased likelihood of HCC recurrence following treatment (128). Thus, strategies aimed at activating NK cells have been investigated, including the transfusion of CAR-NK cells or compounds that promote NK cell function. Among these approaches, CAR-NK-92 cells specifically targeting glypican-3 (GPC3) are effective in combating tumors (129). Cytokines such as IL-12, IL-15, and IL-18 enhance the anti-tumor capabilities of NK cells *in vivo*, leading to the suppression of liver tumorigenesis (126). Furthermore, the antifungal agent lomofungin was found to decrease the activity of soluble MICA, potentially augmenting the cytotoxicity of NK cells against HCC (130). Recent evidence has shown that combining NK cell immunotherapy with irreversible electroporation (IRE), a non-thermal method of tissue ablation, results in enhanced clinical outcomes, including improved progression-free and overall survival, compared with IRE ablation alone in patients with liver cancer (131). These results suggest that adoptive NK cell transfer in combination with diverse strategies holds promise as a beneficial approach for treating advanced HCC.

Pancreatic cancer is a highly aggressive disease with a predicted future increase in its incidence. Similar to CAR-T cells, CAR-NK cells have been tested for their ability to target this disease. In a mouse model of metastatic humanized pancreatic cancer, cord blood-derived NK cells transduced with an anti-prostate stem cell antigen (PSCA) CAR construct containing a soluble form of IL-15 showed efficacy (132). Additionally, in an individual with pancreatic cancer and liver metastasis, anti-ROBO1 CAR NK-92 cells showed minor adverse events and stable disease status for a certain period (78). Clinical trials testing the feasibility of CAR-NK cells or allogeneic NK cell infusion for the treatment of solid tumors are possible strategies (133).

#### Metastatic tumors

Metastasis increases with higher tumor stage and is a significant contributor to cancer-related deaths (134). Metastasis is a complex process and its occurrence is influenced by various factors, including the composition of the tumor microenvironment and the plasticity of cancer cells. Cancer cells undergo several steps during metastasis, such as invasion and dissemination, during which they break away from the primary tumor site. These cells then enter the bloodstream or lymphatic vessels, spread to other parts of the body, and extravasate from the circulation. These processes are known as circulation and colonization, respectively. Afterwards, the cells may enter a dormant state before forming new tumors (135). Studies have shown that polyclonal clusters of cancer cells have better survival and colonization capabilities than monoclonal or single clusters (136–138). Polyclonal clusters are heterogeneous, making them difficult to control. Furthermore, CTCs that are likely to metastasize or respond to treatment can be detected in the blood and protected by platelet adhesion, preventing detection and elimination by NK cells (139).

Accordingly, multiple studies have reported that NK cell-based therapy can potentially target metastatic cancers by eliminating disseminated cancer cells in the circulation or those deposited in other organs (140–142). To understand the mechanism of immune surveillance during the metastatic progression of cancer, it is essential to analyze the characteristics of cancer cells during this process. Initially, invasive cells express keratin-14 (K14) and p63, leading to collective invasion (143). K14 is a basal epithelial marker critical for metastasis. K14-positive cells evade immune surveillance (144, 145). Metastatic cells possess genes related to epithelial and mesenchymal cells (146) as well as cell survival (147). Interestingly, monoclonal and polyclonal clusters of cancer cells exert distinct effects on NK cell resistance. Lo et al. (148) engrafted fluorescently labeled mouse mammary cells, either in a mixed or single population, into the mammary fat pad of recipient mice. The recipient mice included wild-type mice, nude mice lacking T cells, and NOD-Rag1^null^IL2rg^null^ (NRG) mice lacking B, T, and NK cells. The results showed that NRG mice had an increased number of monoclonal metastatic lesions, whereas polyclonal clusters were more dominant in other mice. These findings suggest that these effects may be dependent on immune cells. When NK cells are present, monoclonal clusters can be removed; however, polyclonal clusters are resistant to NK cell surveillance (148). These cells may lose K14, while E-cadherin plays a role in cluster metastasis (149). Similarly, non-cluster-forming cancer cells that are sensitive to NK cell killing express lower levels of genes related to cell-cell adhesion but higher levels of genes encoding ligands for NK cell receptor activation (148). Therefore, altering the epithelial features of metastatic cancer cells, in addition to adoptive NK cell transfer, may provide a strategy for targeting metastasis.

Cancer cells that migrate to different parts of the body enter dormancy. This indicates that these cells undergo cell cycle arrest and remain hidden from the immune system (134, 150). When dormant cancer cells become active again and start dividing, they become sensitive to NK cell killing by decreasing the expression of genes related to MHC class I molecules and increasing the expression of genes related to ligands against NK cell-activating receptors (151). In preclinical models, it was found that in mice lacking B, T, and NK cells, dormant cancer cells increased metastasis, whereas in mice lacking T cells, they decreased (152). Although these findings suggest that NK cells can target metastatic cells under certain conditions, the exact mechanisms by which this occurs, particularly the intrinsic pathways of cancer cells, are not yet fully understood.

Despite the evidence that NK cells can target metastatic cancer, several studies have demonstrated that cancer cells reprogram NK cells to support metastasis. Chan et al. (153) showed that tumor-exposed NK (teNK) cells promoted tumor cell colony growth in a 3D co-culture platform. teNK cells exhibit increased TIGIT and KLRG1 expression and upregulated DNA methyltransferases, such as DNMT1, DNMT3a, and DNMT3b, compared to healthy NK cells, indicating that epigenetic control by cancer cells occurs in teNK cells. Similarly, it is well known that TGF-β signaling induces NK cell-derived ILC1 that show higher expression of immune cell exhaustion markers, including CTLA4 and LAG3 (11). Therefore, the tumor microenvironment and cancer cells can alter NK cells to promote metastasis, which could explain why clinical observations do not always show a correlation between increased NK cell numbers and overall survival in patients with cancer (154).

### Expanding the scope of NK cell therapies

Many countries are becoming increasingly aging societies. All tissues, including those of the immune system, become senescent. Systemic immunosenescence can lead to a decrease in the efficient clearance of harmful cells, resulting in an increase in inflammatory or infectious diseases (155). In this section, we discuss the relationship between age-related diseases and the potential use of NK cell therapies.

#### Cellular senescence and its implication in disease

Cancer incidence in humans increases with age (156). Many biological processes that contribute to cancer and aging share characteristics such as telomere attrition, cellular senescence, genomic instability, and inflammation (157). The development of cancer cells can occur through the evasion of apoptosis or senescence or through the accumulation of senescent cells (158). However, the cellular processes involved in aging and cancer are not yet fully understood, and their relationships are complex. Long-lived species have evolved tumor-suppressing mechanisms and cancer-resistant features (159, 160). Cellular senescence can lead to irreversible cell growth arrest (161), typically in response to damage. This plays a critical role in suppressing abnormal cell proliferation and preventing tumor growth. Senescent cells have been shown to trigger an immune response that aids in tumor clearance. In contrast, studies have demonstrated that senescent cells can promote tumor growth, angiogenesis, and invasion by secreting factors such as extracellular matrix components and cytokines, similar to an immunosuppressive tumor microenvironment. In addition, research has shown that aged fibroblasts can drive lung metastasis and therapy resistance in a mouse melanoma model (162). Understanding the complex interplay between aging and cancer may lead to the development of novel strategies for cancer treatment in aging populations.

Senescent cells contribute to inflammation primarily by releasing a senescent-associated secretory phenotype (SASP) (163), and their accumulation can lead to various side effects in tissues, such as fibrosis. For instance, sarcopenia, the loss of regenerative capacity in skeletal muscle with age, can lead to the replacement of muscle tissue with fat and fibrotic tissues (164). Interestingly, a recent study conducted on mice revealed that senescent cells in muscle tissue can induce age-related changes such as inflammation and increased fibrosis, as well as alterations in gene expression (165). Removing senescent cells from tissues promotes tissue regeneration and suppresses muscle inflammation, suggesting potential beneficial effects.

#### Using immune cells for senolytic therapy

Recently, it has been questioned whether aging can be cured, particularly by removing senescent cells using senolytic agents (166–168). Studies in mice have shown that the removal of senescent cells can reverse age-related pathologies (169, 170). Therefore, it has been suggested that methods used for anti-tumor immune cell therapies can be applied to target senescent cells. Immune cells such as NK cells, macrophages, and T cells can recognize senescent cells (171). Furthermore, senescent cells have increased ligands for NK cell-activating receptors such as NKG2D (172). However, senescent cells can also be resistant to immune surveillance owing to immunosenescence, immune cell dysfunction, or alterations in MHC molecules. A previous study indicated that senescent fibroblasts, which are induced either by the natural aging of human skin fibroblasts or exposure to ionizing radiation, exhibit upregulation of HLA-E, a non-classical MHC molecule, and the pro-inflammatory cytokine IL-6. This upregulation inhibits NK cell surveillance (173). The authors proposed that mAbs targeting the inhibitory receptor NKG2A, similar to existing cancer treatments, could be employed as a senolytic strategy to modulate NK cell activity (174).

Senolytics such as dasatinib in combination with quercetin have been widely used to remove senescent cells, and new senolytic treatments are being developed (175, 176). A recent study showed that infusing NK cells with acein, a non-apeptide that secretes dopamine (177), reversed senescent markers in a mouse model (178). Furthermore, researchers have proposed the use of senolytic CAR-T cells that target urokinase-type plasminogen activator receptor (uPAR) in senescent cells. uPAR was initially identified in senescent models, and the effectiveness of uPAR CAR-T cells in animal models of liver fibrosis has been demonstrated (179). These findings suggest that adoptive immune cell therapies that target senescent cells hold promise as potential approaches for the treatment of aging and aging-related diseases.

#### Autoimmune disorders and viral infections

NK cells play a crucial role in directly eliminating virus-infected cells and stimulating antiviral immune responses through IFN-γ production. The number of NK cells is inversely associated with the severity and recurrence of viral infections in humans (180). Chronic viral infections can contribute to tumor progression and autoimmune disorders, and NK cell dysfunction has been implicated in the pathogenesis of these virus-mediated diseases. Notably, the immunomodulatory roles of NK cells have been investigated in various autoimmune diseases such as multiple sclerosis (MS), rheumatoid arthritis, and systemic lupus erythematosus. NK cells can promote dendritic cell (DC) differentiation during adaptive immunity while also exhibiting the ability to eliminate immature DCs, activated macrophages, and T cells (181). Impaired NK cell cytotoxicity has been observed in patients with autoimmune diseases, suggesting its involvement in disease onset. However, the precise mechanism of action must be contextually considered. For instance, in MS patients, an accumulation of CD56^bright^ NK cells was found in the cerebrospinal fluid, and treatment with daclizumab (a humanized anti-IL-2Rα antibody) led to a significant expansion of CD56^bright^ NK cells accompanied by a reduction in CD4 T cells (182). These observations highlight the critical role of functional NK cells in antiviral immunity and the regulation of autoimmune diseases, although investigations into NK cell therapies for these conditions are still in the early stages of development. Notably, several clinical trials exploring NK cell therapy for the treatment of adults with COVID-19 have recently begun (183). These studies indicate the potential use of NK and CAR-NK cells as therapeutic approaches for managing viral infections.

## Genetic engineering of NK cells for adoptive cell therapy

In NK cell clinical trials, a large quantity of cells is used for infusion, ranging from 5×10^6^ to 1×10^8^ CD3^-^CD56^+^ NK cells per kilogram body weight (184). With the development of techniques and success of CAR-T cell therapies, NK cells have become the next generation of genetically engineered immune cell therapies. This involves the transduction of CARs or other genes that stimulate NK cell function. Gene transfer is accomplished using viruses such as lentiviruses, retroviruses, and adeno-associated viruses (185). The CRISPR-Cas9 system can also be used to delete genes associated with NK cell suppression. A recent report demonstrated efficient knockout of genes using a single guided RNAs and Cas9 protein (RNP) nucleofection method, and the edited NK cells were successfully expanded (186). The authors suggested a clinically relevant protocol using cryopreserved PB NK cells, while ensuring the purity and safety of gene-edited cell therapies as the ultimate goals. The following sections present current strategies aimed at genetically engineering NK cells to enhance their functions, including target cell recognition, NK cell persistence, and tumor infiltration.

### Sources of NK cells for adoptive cell transfer

Different sources of NK cells have been used to develop NK cell therapies, including peripheral blood mononuclear cells (PBMC) (187), umbilical cord blood (UCB) (92), NK-92 cells (188), and induced pluripotent stem cells (iPSCs) (189). Although NK-92 cells derived from NK lymphoma offer a valuable research tool for exploring the function of CARs and genetically engineered cells, their effectiveness may be limited due to the absence of CD16 and the need for irradiation before being administered to patients. In contrast, primary NK cells derived from UCB or PB contain not only cytotoxic NK cells but also CD34^+^ hematopoietic stem cells, which can be expanded and differentiated into mature NK cells. Both UCB- and PB-derived NK cells exhibit therapeutic efficacy against leukemia following CAR gene transduction. Notably, UCB-derived NK cells demonstrate higher proliferative capacity than PB-derived NK cells (190). Additionally, cytokine-induced memory-like NK cells sustained by IL-12, IL-15, and IL-18 in primary NK cells have shown promising clinical outcomes in patients with myeloid neoplasms (187). However, the isolation of large numbers of primary NK cells and hematopoietic stem cells is challenging, and purifying NK cells to deplete CD3 and CD19 positive cells is difficult during allogeneic NK cell transfer. By contrast, iPSC-derived NK cells are an unlimited source of homogeneous human NK cells. These iPSCs can be genetically modified to express or deplete specific genes, followed by therapeutic NK cell differentiation. However, safety concerns remain owing to the presence of undifferentiated iPSCs and the complexity of the differentiation protocol (185).

### Enhancing target cell recognition

CARs enhance immune cell function by combining an intracellular signaling domain with an antigen-specific single-chain variable fragment (scFv) that recognizes a specific antigen. There are four to five generations of CARs with increasing complexity of the intracellular domains, whereas the recognition of antigens is dependent on the ectodomains present in each CAR (191). The second generation, which contains either CD28 or 41BB as a costimulatory domain, is effective and widely used. Second-generation CAR-T cells have been utilized in all FDA-approved CAR-T cell therapies to date. CAR-NK cells are also being developed, with promising results reported in 2020 for anti-CD19 CAR-NK cells for the treatment of certain blood cancers, as previously described (92). At least 30 clinical trials of CAR-NK cells using different sources of NK cells are currently underway worldwide (192). CAR-NK cells are being developed to target different diseases, including hematological malignancies (82–85), solid tumors (78–81), and COVID-19 (**Table 1**).

Despite the ability of second-generation CARs to stimulate NK cells such as T cells, researchers have been investigating novel intracellular CAR domains designed for NK cell activation. Adaptor proteins such as DAP10 and DAP12, which recruit PI3K in association with activating NK cell receptors (**Figure 2**), have been investigated as potential replacements for CD3z in CAR-NK cells (193, 194). Furthermore, NKG2D-engineered CAR (NKG2D-DAP10-CD3z)-NK cells, which targets NKG2D ligands in tumor cells, have been tested (195). Notably, iPSC-derived NK cells were used to test various CAR constructs, and the results showed that NKG2D is a transmembrane domain, followed by 2B4 and CD3z in iPSC-NK cells, producing optimized activity (189). Similarly, DNAM1 expression was found to be more effective than that of CD28 as an intracellular domain in anti-GPC3 CAR-NK-92 cells (196). A better understanding of the mechanisms underlying NK cell biology may lead to the development of novel CAR-NK cell therapies.

### Augmenting NK cell persistence

In addition to CARs, various genes have been investigated to enhance the persistence and function of NK cells *in vivo*. Expression of the membrane-bound form of IL-15 in human PB-derived NK cells has been shown to improve NK cell survival and *in vivo* anti-tumor activity (197). Anti-CD19 CAR, IL-15, and inducible caspase-9-based suicide genes were introduced into CB-derived NK cells, resulting in the extended survival of modified NK cells in a mouse model (198). Scalable and uniformly edited NK cells can be generated from iPSCs. Quadruple-gene-modified iPSC-derived NK cells, called FT576, were created by Cichocki et al. (199) for the treatment of MM by incorporating anti-BCMA CAR (200), a non-cleavable version of the CD16a Fc receptor (201, 202), and a membrane-bound IL-15/IL-15R fusion molecule. These modified NK cells can target BCMA-expressing tumor cells and display improved ADCC and *in vivo* persistence. Additionally, CD38 has been knocked out in NK cells (203), enabling a combination approach with an anti-CD38 mAb for the treatment of relapsed or refractory MM (204).

CRISPR-Cas9 gene editing has been used in many studies to delete negative regulators in NK cells. For example, iPSC-derived NK cells have been edited to remove the cytokine-inducible SH2-containing proteins (CIS), encoded by CISH, resulting in potential IL-15-dependent NK activation (205). Tumor necrosis factor-α (TNF-α)-induced protein-8 like-2 (TIPE2) negatively regulates the immune response of NK cells against tumors. This discovery led to the development of a novel approach involving the use of TIPE2-deficient NK cells, which could potentially enhance the effectiveness of cancer treatment in combination with CISH knockout (206). In animal models, when TIPE2-defecient NK cells are transferred to the host, they activate the IL-15-mTORC1 pathway, leading to improved antitumor activity and providing support for T cell-based immunotherapy (207).

### Improving tumor infiltration and NK cell activation in solid tumors

Genetic disruption of NKG2A has been used to modulate inhibitory signaling in NK cells, resulting in superior tumor control in a mouse model (208). In the tumor microenvironment, adenosine A2A receptor is knocked out in CAR-NK cells to counteract the immunosuppressive effects of adenosine (209). Deletion of TGF-β receptor 2 in CB-derived NK cells (210) or downregulation of TGF-β-induced miR-27a-5p has been used to block TGF-β signaling and improve NK cell function (211). In MM, the therapeutic mAb daratumumab targets CD38, which is expressed by both malignant and NK cells, leading to fratricide. To overcome this issue, CRISPR-modified CD38 knockout NK cells have recently shown improved efficacy (212, 213).

Adoptive NK cell therapy has limited efficacy against solid tumors because of the immunosuppressive tumor microenvironment. To improve the targeting of solid tumors, NK cells have been engineered to overexpress chemokine receptors such as CXCR1, CXCR2, CXCR3, CCR7, and CXCR4 in various animal models of cancer (214–218). In addition, the overexpression of a high-affinity dominant-negative TGF-β receptor (TGF-β DNRII) has been used to inhibit TGF-β signaling (219, 220). Clinical trials on patients with prostate cancer have shown the feasibility of expressing TGF-β DNRII and T cells in combination with the antiprostate-specific membrane antigen CAR (221). Another approach involves targeting the inhibitory receptors on NK cells via viral transduction. For instance, human PB NK cells were transduced with a retrovirus to express NKG2A protein expression blocker (PEBL), which abrogates NKG2A expression (208). The PEBL construct contained an anti-NKG2A scFv linked to four different endoplasmic reticulum retention domains, which resulted in enhanced NK cell cytotoxicity and *in vivo* anti-tumor activity.

## Therapeutic approaches to harness NK cell activation

**Figure 3:**
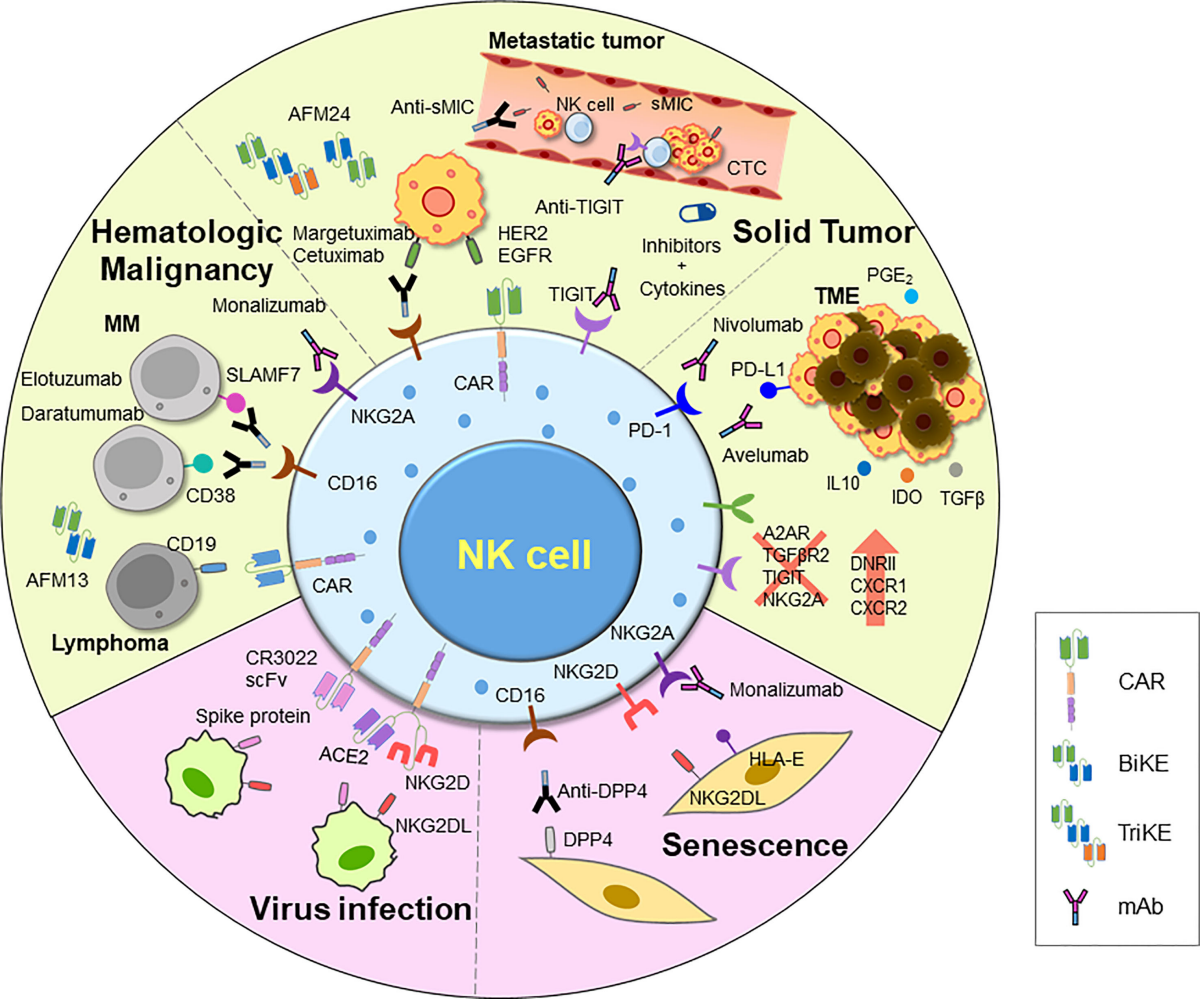
An overview of current and emerging approaches for harnessing NK cell activity. Adoptive transfer of NK cells has demonstrated efficacy in treating tumors, and various strategies have been employed to further improve their function. These include introducing CARs, chemokine receptors, and other modifications via genome editing using CRISPR-Cas system. Additionally, ADCC has been shown to efficiently induce NK cell killing activity. Clinical trials are underway for various therapies for inducing ADCC, including mAbs, BiKEs, and TriKEs. Researches on developing TME inhibitors and cytokines to enhance cell activity, and combinations of these agents with NK cells or other treatments are also being explored. NK cell therapy is also being investigated for novel indications such as viral infections and aging, using therapeutic NK cells to eliminate damaged cells. *CAR*, chimeric antigen receptor; *mAb*, monoclonal antibody; *BiKE*, bi-specific killer engager; *TriKE*, tri-specific killer engager; *sMIC*, soluble MHC I chain-related molecules A and B; *DPP4*, dipeptidyl peptidase-4; *IDO*, indoleamine-pyrrole 2,3-dioxygenase; *TME*, tumor microenvironment; *DNRII*, dominant-negative TGF-β receptor 2.

Immunotherapeutic approaches to enhance NK cell function, such as monotherapy and combination with other strategies, have rapidly developed. This section covers various types of NK cell activators, including those specifically designed based on NK cell biology and those that have been found to activate NK cells. These agents can be combined with NK cell or CAR-NK cell therapies. Various adoptive NK cell therapies in different combinations are presented in **Table 2**. An overview of current NK cell therapies and targetable diseases is summarized in **Figure 3**.

**Table 2:** A list of current strategies of adoptive NK cell therapy in combination with other treatments (chemotherapy, radiation therapy and immunotherapy).

Cell type	Combination (Strategy)	Target Disease	Clinical Trial Phase	Trial Status	Clinical Trial register	Ref
NK-92	N/A	Stage IIIb and IV Merkel cell carcinoma	Phase 2	Unknown	NCT02465957	
NK-92	Chemotherapy	Pancreatic cancer	Phase 1,2	Unknown	NCT03136406	
haNK	Chemotherapy Radiation therapy	Pancreatic cancer	Phase 1,2	Unknown	NCT03329248	
haNK	Chemotherapy Radiation therapy	Squamous cell carcinoma	Phase 1,2	Unknown	NCT03387111	
haNK	Chemotherapy Radiation therapy	Triple-negative breast cancer	Phase 1,2	Active, not recruiting	NCT03387085	
haNK	Chemotherapy Radiation therapy	Metastatic colorectal cancer	Phase 1,2	Active, not recruiting	NCT03563157	
PB-NK	N/A	Plaque psoriasis	Phase 1	Completed	NCT03894579	
PB-NK	N/A	Cancers	Phase 1	Completed	NCT00569283	(75)
PB-NK	N/A	Malignant solid tumor	Phase 2	Completed	NCT02853903	
PB-NK	N/A	Recurrent childhood medulloblastoma/ Ependymoma	Phase 1	Complete	NCT02271711	
PB-NK	N/A	Hepatocellular carcinoma	Phase 2	Completed	NCT02854839	
PB-NK	N/A	Small cell lung cancer	Phase 2	Unknown	NCT03410368	
PB-NK	Immunotherapy (hu3F8 antibody)	Neuroblastoma	Pahse 1	Active, not recruiting	NCT02650648	
PB-NK	Immunotherapy (PD-L1 antibody)	Non-small cell lung cancer	Phase 2	Unknown	NCT03958097	(118)
PB-NK	Chemotherapy	Refractory/Metastatic/Recurrent/Advanced Cancer Unresectable carcinoma	Phase 1	Active, not recruiting	NCT03941262	
PB-NK	Chemotherapy	Nasopharyngeal cancer Head and neck squamous cell carcinoma	Phase 1,2	Unknown	NCT02507154	(222)
PB-NK	Chemotherapy	Breast cancer Gastric cancer	Phase 1,2	Unknown	NCT02030561	
PB-NK	Chemotherapy	Gastric cancer	Phase 1,2	Unknown	NCT02805829	
PB-NK	Chemotherapy	Non-small cell lung cancer	Phase 2	Unknown	NCT02734524	
PB-NK	Chemotherapy	Biliary tract cancer	Phase 1,2	Completed	NCT03937895	
PB-NK	Chemotherapy Radiation threapy	Colon cancer stage IV	Phase 1,2	Unknown	NCT03329664	
CB-NK	Chemotherapy	Cutaneous melanoma Lip and oral cavity carcinoma Malignant neoplasm	Phase 1	Recruiting	NCT03420963	
CB-NK	Chemotherapy	Ovarian carcinoma Fallopian tube carcinoma Primary peritoneal carcinoma	Phase 1	Recruiting	NCT03539406	
NK-92	Chemotherapy Immunotherapy Radiation therapy	Myelodysplastic syndrome Leukemia Lymphoma Multiple myeloma	Phase 2	Recruiting	NCT02727803	
NK-92 CB-NK	Chemotherapy Immunotherapy Radiation therapy	Myelodysplastic syndrome Leukemia Lymphoma Multiple myeloma	Phase 2	Recruiting	NCT02727803	
PB-NK	N/A	Acute lymphoblastic leukemia Complete hematologic remission Persistent/Recurrent minimal residual disease	Phase 1	Active, not recruiting	NCT02185781	
PB-NK	N/A	Asymptomatic multiple myeloma	Phase 2	Completed	NCT01884688	
PB-NK	N/A	NK cell mediated immunity	Phase 1	Unknown	NCT03662477	(119)
PB-NK	N/A	Acute leukemia	Phase 1,2	Completed	NCT03669172	(77)
PB-NK	N/A	Acute myeloid leukemia Myelodysplastic syndromes	Phase 1,2	Recruiting	NCT03300492	
PB-NK	N/A	Acute myelogeneous leukemia Acute lymphoblastic leukemia	Phase 1,2	Completed	NCT01795378	(75)
PB-NK	Chemotherapy	Multiple myeloma	Phase 1	Completed	NCT02481934	(87)
PB-NK	Chemotherapy	Chronic myeloid leukemia Pancreatic cancer Colorectal cancer Multiple myeloma Non-small cell lung cancer	Phase 1	Completed	NCT00720785	
PB-NK	Chemotherapy	Metastatic melanoma Metastatic kidney cancer	Phase 2	Completed	NCT00328861	
PB-NK	Chemotherapy	Acute myeloid leukemia	Phase 1,2	Unknown	NCT02809092	(76)
PB-NK	Chemotherapy	Acute myeloid leukemia	Phase 1,2	Terminated	NCT01898793	(223)
CB-NK	Chemotherapy	Lymphoma	Phase 2	Completed	NCT03019640	

N/A, Not applicable; haNK, high affinity NK cell, NK-92 cells engineered to express the high affinity CD16; PB-NK, peripheral blood derived NK cell; CB-NK, cord blood derived-NK cell.

### Antibodies

#### Antibodies for ADCC responses

NK cells exert their effector function through the ADCC-mediated killing of IgG1- or IgG3-opsonized target cells, which are recognized by CD16 in NK cells. Therapeutic antibody development often excludes IgG3 because of its long hinge region and polymorphic nature (224). Several ADCC-inducing antibodies such as rituximab (anti-CD20), cetuximab (anti-EGFR), and trastuzumab (anti-HER2) for CLL, colorectal cancer, and breast cancer, respectively, have been developed and approved by the FDA for tumor treatment (222). Notably, patients with the CD16 polymorphism at position 158F (phenylalanine) have a limited response rate owing to their low affinity for the Fc region of antibodies, in contrast to those with the 158V (valine) variant, which exhibits a high affinity (225, 226). To overcome this problem, the development of mAbs that induce ADCC has focused on improving affinity of low-affinity variant. Elotuzumab and daratumumab target SLAMF7 and CD38, respectively, and induced robust ADCC in myeloma cells (204, 227). Margetuximab, an anti-HER2 antibody, also shows improved binding to low-affinity CD16 and has received approval from the US FDA (228). Avelumab, an IgG1 antibody against PD-L1, provides the additional benefit of inducing checkpoint inhibition in addition to ADCC (229, 230). Furthermore, anti-MICA antibodies have been suggested to enhance NK cell cytotoxicity via NKG2D and ADCC engagement (231).

#### NK cell engagers

The use of bi- and tri-specific killer engagers (BiKEs and TriKEs, respectively) in therapeutic approaches is similar to that of ADCC-inducing antibodies. These molecules facilitate interactions between effector and target cells, leading to the activation of NK cells through CD16 engagement. Unlike Fc-targeted antibodies, BiKEs and TriKEs only target CD16 via Fv fragments, thereby reducing the off-target effects. AFM13, a BiKE targeting CD30 for B- and T-cell lymphomas (232), has been granted orphan drug designation for the treatment of peripheral T-cell lymphoma (NCT4101331, NCT04074746) (233). Another BiKE, AFM24, that targets EGFR in solid cancers, has also been studied (NCT04259450). During the development of TriKEs, moieties such as IL-15, which enhance NK cell function, can be incorporated in addition to BiKEs. Studies have investigated the effectiveness of TriKEs containing IL-15 against several tumor antigens, such as CD133, CD19, CLEC12, and CD33 (234–236). TriKEs also target additional NK cell receptors, such as NKp46 (237).

#### Immune checkpoint inhibitors

Currently, immune checkpoint inhibitors (ICIs) such as anti-PD1, PD-L1, and CTLA4 antibodies have shown success in stimulating the immune system to eradicate tumors (238). The main mechanism of action is thought to be prevention of T cell exhaustion. Nevertheless, it has been suggested that NK cells could also benefit from ICIs (239, 240). By activating NK cells through the ADCC-mediated induction of PD-L1 on the tumor cell surface, combining ICIs with ADCC immunotherapy may have a synergistic anti-tumor effect (238). Targeting other immune checkpoints such as LAG3, TIM3, and TIGIT is currently being investigated because these receptors are also expressed on tumor-infiltrating NK cells (241, 242). Previous studies have demonstrated that TIGIT antibody can increase NK-mediated antitumor response (113).

Efforts have been made to develop ICIs that are specific to NK cells, owing to their possession of various inhibitory receptors that could potentially act as immune checkpoints. Lirilumab, a KIR antibody, was developed to target the HLA-C-specific family of KIR2D receptors, which can be either inhibitory or activating (243). However, the clinical use of lirilumab alone has not resulted in significant responses. Therefore, combination strategies are being explored (NCT02813135). Another potential target for NK-specific immune checkpoint inhibition is NKG2A, as its ligand, HLA-E, is often upregulated in tumors and senescent fibroblasts (173, 244). Monalizumab (IPH2201), an NKG2A antibody, is currently being investigated in clinical trials in combination with other agents for the treatment of solid tumors, such as in head and neck cancer and breast cancer (NCT04590963, NCT02643550, NCT04307329, and NCT02671435). Notably, inhibitory KIR plays a crucial role in NK cell education and licensing, and NKG2A is important for maintaining NK cell immune tolerance; thus, caution should be exercised when targeting these receptors (245).

### Other NK cell activating agents and strategies

There are several ways to enhance the activity of NK cells against tumor cells, including pharmacological interventions or the use of cytokines. Immunomodulatory imide drugs (IMiDs) such as lenalidomide and pomalidomide, which interact with cereblon (CRBN), can activate NK cells via CRBN-dependent or independent mechanisms (246, 247). IMiDs activate ZAP70 and increase granzyme B expression in NK cells (248). In addition, IMiDs make tumor cells more susceptible to NK cell killing by upregulating ligands for NKG2D, DNAM1, MICA, and PVR in tumor cells (249). Bortezomib, a proteasome inhibitor, has been shown to have similar effects on tumor cells, including downregulation of MHC-I expression (250). Other pharmacological interventions that increase the vulnerability of cancer cells to NK cell killing have also been reported. BH3 mimetics enhance NK cell-mediated killing by inducing mitochondrial apoptosis in target tumor cells (251). ONC021, an orally administered antitumor agent, activates TNF-related apoptosis-inducing ligand (TRAIL) and stimulates NK cells at the tumor site, leading to reduced metastasis. Clinical trials for ONC021 are currently underway (NCT02525692) (252).

The direct stimulation of NK cells has also been extensively studied, particularly in the context of the tumor microenvironment, where immune cells are often suppressed. For example, HIF1α expression induced by a hypoxic tumor microenvironment can inhibit NK cell activity, but inhibition of HIF1α or using *Hif1α* ^-/-^ NK cells has been shown to potentiate NK cell activity against tumors in *in vitro* and *in vivo* experiments (253). Various pharmacological inhibitors against the TGF-β signaling pathway have been tested in preclinical and clinical studies. It has been shown that the TGFβRI kinase inhibitor galunisertib (LY2157299) enhances NK cell-mediated anti-tumor activity (254). On the other hand, ADAM17, a metalloproteinase that cleaves CD16 on the surface of NK cells, can suppress ADCC responses (255). Although ADAM17 inhibitors have been proposed to enhance ADCC in NK cells, the potential advantage of using ADAM17 inhibitors remains a matter of ongoing debate (256).

The cGMP-AMP synthase (cGAS)-stimulator of interferon gene protein (STING) pathway is crucial for immune cell activation via the production of type I IFNs (IFNα and IFNβ) (257). Interestingly, cyclic dinucleotides (CDNs), which are STING agonists, have been shown to activate NK cells (258). NK cell activation appears to be driven by increased IL-15 production in dendritic cells treated with CDNs, in response to increased type I IFNs. Notably, several STING agonists, including CDNs and small molecules, are currently undergoing clinical testing for tumor treatment (259).

Numerous cytokines have been identified as stimulators of NK cell proliferation and activation and cytokine mixtures are being studied to develop adoptive NK cell maturation strategies for cancer treatment. Furthermore, efforts are being made to develop cytokines that can be used as direct cancer treatments, including IL-2 (260), IL-15 (261), IL-12 (262), IL-18, and IL-21 (263). However, most of these cytokines have toxic adverse effects and limited efficacy when used as natural proteins. Consequently, novel approaches are being explored such as combining cytokines with other therapeutics or improving their delivery to target tissues. Among these cytokines, IL-15 potentiates NK and CD8^+^ T cell activity. Clinical trials are currently underway for heterodimeric fusion (hetIL-15) packaged in extracellular vesicles (264) and the superagonists IL-15 and N-803 (223, 265). Delivery methods for IL-12 such as direct intratumoral gene delivery or plasmids in combination with PD-1 inhibitors have also been tested (266). Furthermore, a modified form of the proinflammatory member of the IL-1 cytokine family, IL-18, has been suggested (267).

## Conclusion

There are multiple subsets of NK cells and their activities can vary within each organ because of their dynamic nature. Exploiting the cytotoxic efficacy of NK cells through therapeutic strategies, such as adoptive NK cell therapy and BiKEs, shows great promise. Although safety is advantageous in adoptive NK cell therapy, optimizing combination approaches by understanding NK cell-specific mechanisms may enhance their efficacy. For example, combining cytokine-induced memory-like NK cells (187) with CAR (268) or BiKE (269) expression results in improved responses. Novel indications for efficient NK cell therapies, such as for virus infection, fibrosis, and aging, are emerging. With the advent of immune surveillance against abnormal cells, some researchers have shifted their focus to targeting senescent cells. Further investigations on NK cell immunity and cell-cell interactions can greatly support these new phenomena. Advances in genome editing, immune cell expansion, stem cell biology, and antibody design technologies are crucial for the progress in this field. Thus, NK cells have great potential as novel therapeutic agents against various diseases.

## Author contributions

J-YN wrote the manuscript; ES, SB, JK, and TP produced figures and tables; ES, S-RY and HJ edited the manuscript. J-YN supervised the overall project and edited the manuscript. All authors contributed to the article and approved the submitted version.
